# Fingernails changes associated with chemotherapy in breast cancer: Muehrcke’s lines

**DOI:** 10.1002/ccr3.1642

**Published:** 2018-07-03

**Authors:** Xiaosong Chang, Peng Zhen, Jian Zeng

**Affiliations:** ^1^ Department of Oncology Chifeng 220 Hospital Chifeng Inner Mongolia Autonomous Region China

**Keywords:** breast cancer, chemotherapy, fingernails, Muehrcke’s lines

## Abstract

Nail abnormalities are an indicator of systemic disease but are always neglected during clinic visit. Here, we report a rare case of Muehrcke’s lines accompany with normal range serum albumin after chemotherapy, unlike the hypoalbuminemia in most cases, which suggest malnutrition is not the only cause of Muehrcke’s lines.

A 36‐year‐old woman was diagnosed with breast cancer, which is on outer upper quadrant of right side with 2.0 cm × 3.0 cm in size. Modified radical mastectomy was performed. The histopathological report showed invasive ductal carcinoma (moderate‐poorly differentiated) with invasion to adipose tissues and axillary lymph nodes cancer (10/31), _p_T2N3M0, stage IIIc. The patient was treated with cyclophosphamide, doxorubicin, and 5‐fluorouracil chemotherapy in our hospital. After the fifth cycle of chemotherapy, Muehrcke’s lines were presented on the fingernails (Figure 1, arrows). The patient was with normal blood, urine, and feces test results, and particularly total protein, albumin and globulin were within normal range. Five months later, Muehrcke’s lines disappeared. The patient eventually died from tumor relapse and metastasis at age of 40.

**Figure 1:**
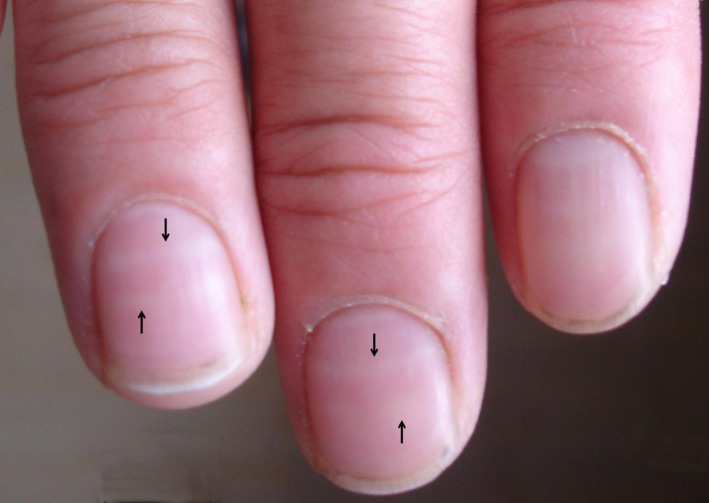
Muehrcke’s lines: two symmetrical, narrow, transverse white lines, which run parallel to the edge of the lunula across the width of the nail.

Muehrcke’s lines are the two symmetrical, narrow, transverse, horizontal, nonpalpable white lines on the fingernails that span the entire breadth of the nail and run parallel to the edge of the lunula. Compression of the digits results in temporary fading of the lines. Because the lines are in the nail bed and do not move with the growth of the fingernails. Normal pink nail bed tissue can be seen between the two white lines and thumb involvement is rare. These features are different from Mees’ lines.^1^, ^2^ The underlying pathogenesis of the lines is incompletely understood but may be caused by vascular compression abnormality of the vascular bed of the nail,^3^, ^4^ or the vascular alteration may reduce the adherence between the distal nail plate and the nail bed.^5^ Muehrcke’s lines^6^ were first reported in 1956 in patients with nephritic syndrome and hypoalbuminemia from other causes. The lines appeared when serum albumin levels were lower than 2.2 g/dL, and disappeared after the serum albumin increased to greater than 2.2 g/dL. Since then, Muehrcke’s lines were reported in patients with malnutrition,^7^ trauma,^5^ and liver disease,^3^ most commonly in patients treated with multiple cytotoxic chemotherapy agents.^8^, ^9^, ^10^, ^11^ A case was reported in 2007^12^ that a 46‐year‐old woman developed Muehrcke’s lines after five cycles chemotherapy with doxorubicin, ifosfamide, and mesna for metastatic sarcoma, the nonspecific finding may be associated with periods of metabolic stress, during which time the body’s ability to synthesize proteins is impaired, the patient died from progressive metastatic disease after 3 months. In our case, Muehrcke’s lines also appeared after five cycles chemotherapy. However, this patients’ Muehrcke’s lines disappeared 5 months later and never came back until death. This case’s clinical features were typical, and the serum protein levels were normal, and there were no history of metabolic or endocrine changes. During the chemotherapy of breast cancer, it is relatively rare to present Muehrcke’s lines, which may be associated with chemotherapy.^11^, ^13^ Therefore, it is necessary to inspect fingernails carefully during physical examinations. Whether the abnormal fingernails findings could predict any pathology changes in the patient with malignant tumors is an interesting topic, and it deserve further work‐up in the future clinical practice.

## AUTHORSHIP

XC: diagnosed the case, took photo and wrote the manuscript. PZ and JZ: had advisory roles in the management of the patient.

## CONFLICT OF INTEREST

None declared.
